# Pedunculated Basal Cell Carcinoma Arising on the Nipple of a 62-Year-Old Man: An Atypical Presentation of the Most Common Skin Cancer

**DOI:** 10.7759/cureus.111301

**Published:** 2026-06-22

**Authors:** Bret Andersen, Peter Lee, Pooja Srivastava, Munir H Idriss

**Affiliations:** 1 Dermatology, Sanford Health, Marshfield, USA; 2 Dermatology, Sanford Health, Eau Claire, USA; 3 Pathology, Sanford Health, Marshfield, USA

**Keywords:** basal cell carcinoma (bcc), dermatopathology, mohs surgery, nipple areolar complex, nipple lesion, non-melanoma skin cancer (nmsc), ­skin cancer

## Abstract

Basal cell carcinoma (BCC) is the most common type of skin cancer, and the greatest risk factor for its development is ultraviolet (UV) radiation exposure related to the sun or tanning. It most commonly presents on sun-exposed areas such as the head and neck, and it commonly has characteristic clinical and dermoscopic findings. This case illustrates a rare presentation of BCC with an unusual location and clinical morphology. The lesion was treated with Mohs micrographic surgery, with no recurrence at 24 months.

## Introduction

Basal cell carcinoma (BCC) is the most common type of skin cancer and represents approximately 80% of non-melanoma skin cancers, with incidence rates as high as 1,019 cases per 100,000 person-years for women and 1,488 cases per 100,000 person-years for men [1]. BCC originates from the epidermal basal cells and is primarily caused by prolonged exposure to ultraviolet (UV) radiation from the sun [2]. Genetic factors, exposure to certain chemicals, and immunosuppression can also contribute [2].

While BCC is generally considered to be a low-grade malignancy with a low risk of metastasis, it can cause significant local destruction and morbidity if left untreated [3]. The clinical management of BCC typically involves surgical excision, Mohs micrographic surgery, destruction, radiation, or topical therapies, depending on the tumor's clinical and pathological characteristics, such as location, size, and histopathologic subtype [4]. Mohs surgery utilizes intraoperative complete margin assessment via histopathologic examination of frozen tissue sections, and it is associated with the lowest recurrence rates for high-risk BCC [4]. Understanding the various presentations of BCC is crucial for early detection and effective treatment, particularly in cases with atypical morphology or location. Herein, we report a case of BCC of the nipple areola complex with an unusual pedunculated clinical morphology.

## Case presentation

**Figure 1:**
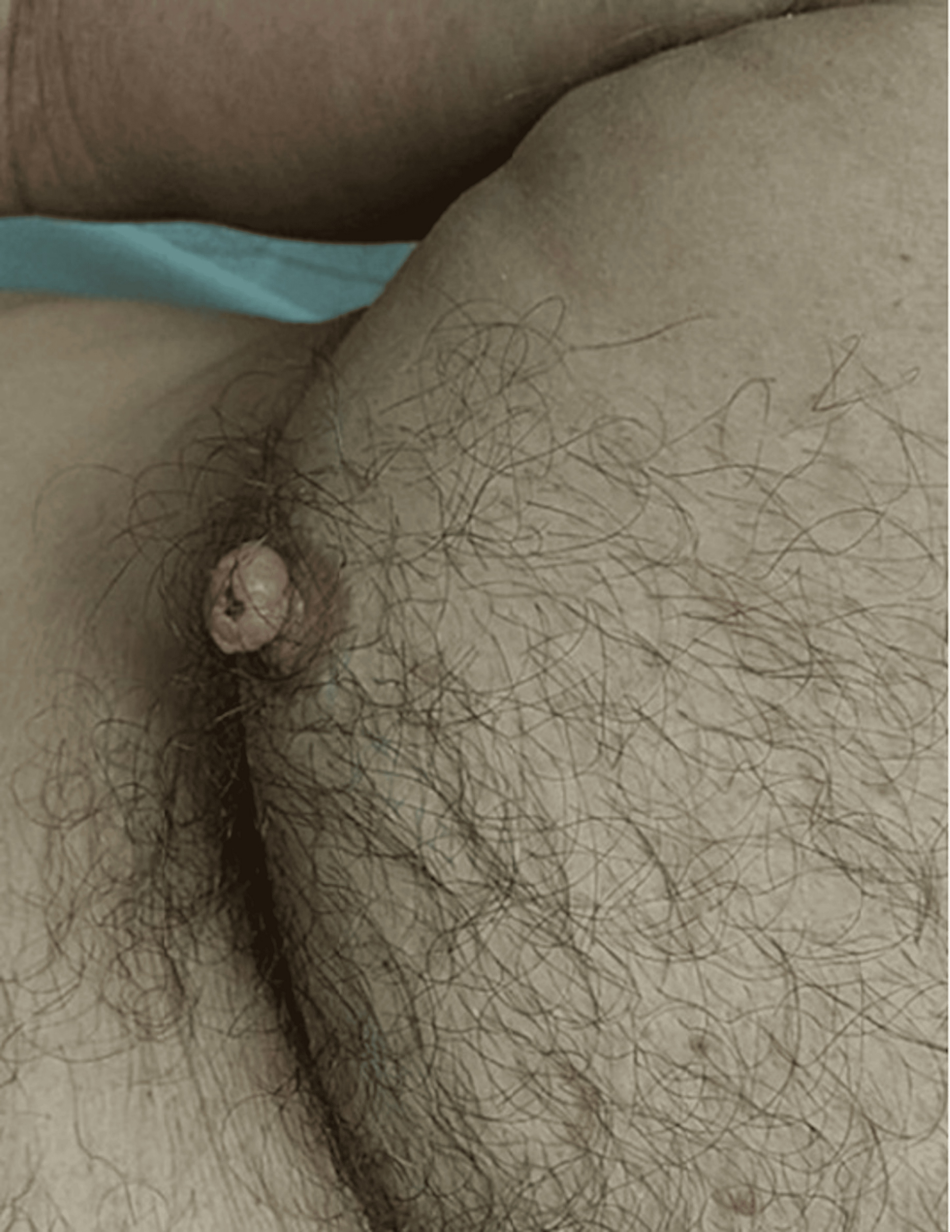
Lesion at initial clinical presentation, a 1.2 cm pedunculated, eroded, and crusted pink nodule of the right nipple.

A 62-year-old male patient with no prior history of malignancy or other skin disease presented to his primary care provider for his annual health maintenance visit, at which time a lesion of the right nipple was noted, as shown in Figure 1.

**Figure 2:**
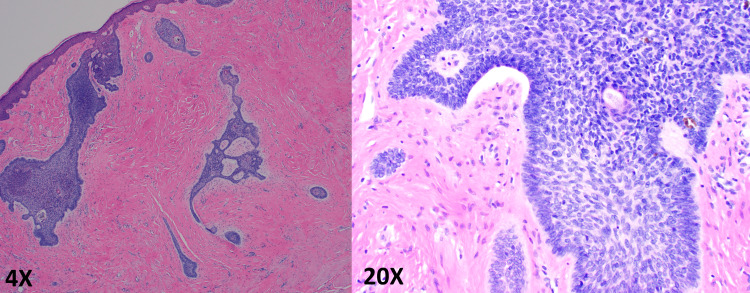
Biopsy histopathology showing basal cell carcinoma. Emanating from the basal layer of the epidermis are islands of basophilic tumor cells representing basal cell carcinoma, with peripheral palisading of the nuclei, associated fibromyxoid stroma, and stromal retraction. The sections show dense fibrous stroma with bundles of smooth muscle, suggestive of skin from the nipple region (hematoxylin & eosin stain, 4x (left pane) and 20x (right pane) magnification).

The lesion had been present for six to eight months, with associated bleeding and chronic irritation in the area. The physician noted concern for an inflammatory process, such as irritant contact dermatitis, versus a possible breast cancer. They referred the patient to dermatology, where the lesion was noted to be a 1.2-cm pedunculated pink nodule of the right nipple with surface erosion and crusting. The lesion otherwise showed no characteristic findings on dermoscopic examination. Given suspicion for a neoplastic process, biopsy of the lesion was offered, though declined in favor of two weeks of conservative topical therapy with clobetasol and mupirocin ointments. After two weeks, the patient returned to the clinic with no clinical change. The lesion was biopsied, and the pathology findings are shown in Figure 2.

**Figure 3:**
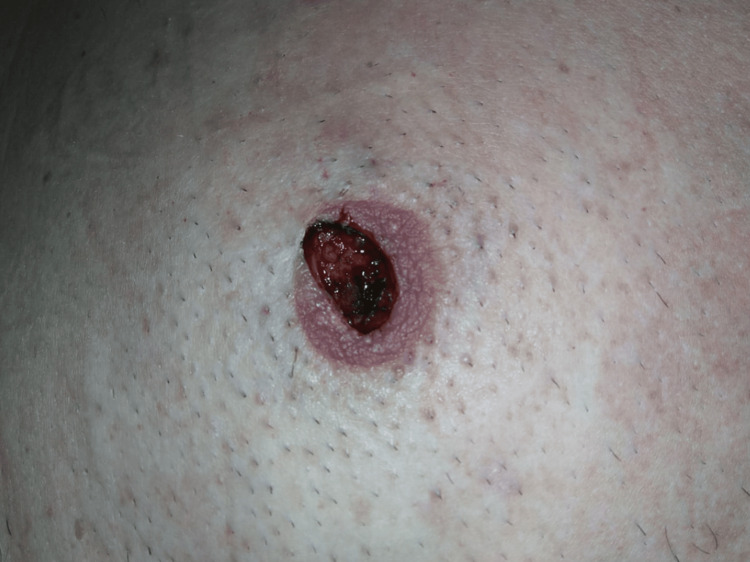
Post-Mohs surgical defect, measuring 2.0 cm by 1.3 cm.

The findings indicated basal cell carcinoma, nodular and superficial subtypes, extending to the margins, and treatment options were considered and discussed, including Mohs micrographic surgery, destruction procedures, radiation, and topical therapy. The lesion received a Mohs Appropriate Use Criteria score of 8, and the patient was referred to Mohs micrographic surgery for definitive surgical treatment [5]. The tumor was debulked via shave excision to confirm diagnosis and location. Then, a first Mohs stage was taken via full-thickness excision around the clinical extent of the lesion. The tissue was processed as a frozen specimen, mapped, and color-coded at the margins. A microscopic tumor was found at the subcutaneous margin. A second Mohs stage was taken in the area of positivity, processed as a frozen specimen, and a tumor-free margin was noted. The final defect extended into the fascia and glandular tissue and measured 2.0 cm by 1.3 cm, as shown in Figure 3.

A complex layered linear closure was performed. The patient had no evidence of recurrence on follow-up at 24 months postoperatively.

## Discussion

Herein, we present an unusual location and morphology of BCC in a patient with otherwise unremarkable clinical characteristics. BCC of the nipple represents a rarity, with a comprehensive 2016 literature review documenting 55 cases reported from 1893-2016 [6]. BCC of the nipple occurs more often in men (63.6%), as seen in this case, and this has been hypothesized to be related to greater UV exposure of the chest in men as compared to women [6].

Clinical appearance of BCC often correlates with histologic subtype. Nodular BCC, the most common histologic subtype, often appears as a pink or translucent papule with telangiectatic vessels and a rolled border [7]. Other forms with characteristic appearances include superficial BCC (scaly plaque), morpheaform (scar-like) BCC, and pigmented BCC. Findings on dermoscopy include arborizing telangiectatic vessels, blue-gray ovoid nests of pigment, leaflike areas, ulceration, crusting, and lack of a pigment network [8]. Of note, this case presented with ulceration and crusting, otherwise without such characteristic clinical or dermoscopic findings noted. One report suggests a so-called “large black web” finding on dermoscopy in BCC of the nipple, with pigment avoiding the hair follicles, though this was also not seen in the present case [9].

As noted by Chun et al., 50% of previously reported BCCs of the nipple-areolar complex presented as plaques, 23.5% as nodules, 17.5% as papules, 5.9% as macules, and 2.9% as patches [6]. In contrast, our case presented as an exophytic and pedunculated mass, unrepresented in the previously reported literature. On histology, previously reported BCCs of the nipple-areolar complex were most frequently nodular (42.9%), followed by superficial (30.9%), mixed (16.7%), fibroepithelioma of Pinkus (9.5%), and pigmented (26.2%) [6]. Of the mixed subtypes, 42.9% were noted to have aggressive features on histology, such as infiltrative (42.9%) or micronodular (14.3%) patterns [6]. Our case represented a mixed pattern of nodular and superficial subtypes, without aggressive features on histology.

The referring provider’s concern limited to a possible inflammatory process or breast cancer highlights the lack of general awareness of BCC of the nipple. In our case, the differential diagnosis may include Paget’s disease, squamous cell carcinoma, amelanotic melanoma, breast cancer, nipple adenoma, neuroendocrine tumor, or other adnexal neoplasms. Of note, all these concerns warrant a biopsy for diagnosis. Regarding breast cancer in particular, there is a rare aggressive form, basaloid carcinoma of the breast (BCB), which is uniformly triple negative for estrogen receptor, progesterone receptor, and HER2/neu amplification and which carries a poorer prognosis. It may histologically mimic cutaneous basal cell carcinoma or other basaloid neoplasms, though it lacks the peripheral palisading or dermal mucin typically seen in BCC. Immunohistochemistry can be helpful in distinguishing BCB from BCC and other basaloid neoplasms. Such stains include BerEP4, EMA, Bcl-2, GATA3, CK7, and CK17 [10,11]. The overall clinic picture is also important in distinguishing BCB from BCC, and our patient was without other consistent features or a history of known breast cancer.

While metastasis of BCC is rare at less than 0.05%, two reviews estimating metastases in BCC of the nipple ranged from 9.1 to 11.5%, hypothesized as being due to increased lymphatics and capillaries in the subareolar plexus [3,12]. Thus, while prompt diagnosis and effective treatment are always of high importance in BCC in order to minimize the surgical defect and thus necessary repair, this may be increased in cases involving the nipple. As seen in this case, the defect can be sizeable and can extend into adjacent important structures, even with the utilization of relatively tissue-sparing techniques with margin control, such as Mohs micrographic surgery. Given reported metastasis rates of 9-12%, sentinel lymph node biopsy can be considered or discussed with the patient in select cases.

## Conclusions

This case demonstrates an uncommon presentation of basal cell carcinoma of the nipple, without characteristic morphology or dermoscopic findings that might otherwise aid in clinical diagnosis. While the nipple is a less common location of BCC, it is important to recognize the breadth of possible presentations, as alternative diagnoses may lead to inappropriate referrals, workup, or treatments. These lesions, as well as other lesions that are clinically atypical or persistent despite appropriate therapy, warrant prompt biopsy for diagnostic confirmation, most often with subsequent timely and definitive surgical treatment, such as with Mohs micrographic surgery.
